# Fabrication of Composite Material by Directly Printing Resin on Aluminum Foam by 3D Printer

**DOI:** 10.3390/ma17051124

**Published:** 2024-02-29

**Authors:** Yoshihiko Hangai, Reiji Yamazaki, Takaaki Suzuki, Nobuhiro Yoshikawa

**Affiliations:** 1 Graduate School of Science and Technology, Gunma University, Kiryu 376-8515, Japan; 2 Institute of Industrial Science, The University of Tokyo, Tokyo 153-8505, Japan

**Keywords:** cellular materials, composite, 3D printing, foam, X-ray CT

## Abstract

Aluminum foam has relatively low tensile and flexural strengths because it is composed of many pores with thin cell walls. One method of strengthening aluminum foam is to fabricate a composite material with a dense lightweight resin. In this study, the fabrication of composite materials by directly printing resin on an aluminum foam surface using a 3D printer was attempted. The resin was directly printed on both heated and unheated aluminum foam. It was shown that composite materials consisting of aluminum foam and resin can be fabricated by directly printing resin with a 3D printer on both heated and unheated aluminum foam. The resin was softened during the printing process in the case of directly printed resin on heated aluminum foam, allowing more resin to penetrate into the pores than in the case of directly printed resin on unheated aluminum foam. In addition, it was shown that resin can be directly printed on the aluminum foam with a high bonding strength, as a large amount of resin penetrated into the pores, resulting in an anchor effect. That is, composite materials consisting of aluminum foam and arbitrary-shaped resin with relatively high bonding strength can be fabricated when a large amount of resin is allowed to penetrate into the pore.

## 1. Introduction

Aluminum foam is a lightweight material with excellent shock absorption and heat insulation properties, and it is expected to be used for automotive components and building materials [1,2]. However, it also has the problem of low tensile and flexural strength because it is composed of many pores with thin cell walls. Composite materials consisting of aluminum foam and dense surface sheets are an effective solution to this problem [3,4,5]. Metal sheets are often used as dense sheet materials [3,4,5,6,7]. In recent years, composite materials of aluminum foam and lightweight resin have also been attempted to meet the trend toward multi-materials [8,9,10,11]. Most of them use adhesives [12,13], but methods that require no adhesive have also been proposed, as described below. For example, there are some attempts to thermocompress resin sheets onto heated aluminum foam such as the combination of aluminum alloy sheets with porous layers and Polyamide-6 [14,15], aluminum alloy sheets with porous layers and carbon fiber-reinforced thermoplastic [16], and aluminum foam with polycarbonate [17,18]. Composite materials of open-cell aluminum foam with interpolation of polyurethane, silicone polymer rubber, and epoxy resin have also been attempted [19,20]. There have also been attempts to fabricate composite materials by applying epoxy resin directly onto the aluminum foam surface followed by curing the resin [21,22,23,24], and by filling the aluminum foam surface with polyamide 12 using selective laser melting [25]. Furthermore, the fabrication of composite materials consisting of open-cell nickel foam and polymethyl methacrylate has been attempted using friction stir incremental forming [26]. Friction welding of plastic sheets and aluminum foam has also been attempted [27,28,29].

In recent years, 3D printers have made remarkable progress [30,31,32,33,34,35,36,37], and designed shapes on a computer can be directly formed by a 3D printer. In this study, we attempted to fabricate a composite material by directly printing resin on an aluminum foam surface using a 3D printer. It is expected that the composite material consisting of aluminum foam and arbitrary-shaped resin can be fabricated by a 3D printer. Especially in the case of small-quantity, high-mix production, this method is considered effective. In conventional studies of fabricating composite materials consisting of aluminum foam and resin, thermocompression bonding was often used to soften the resin and facilitate its penetration into the pores. In this study, resin was directly printed on heated aluminum foam. In addition, a sample in which the resin was directly printed on aluminum foam without heating was also fabricated for comparison. The obtained samples were subjected to X-ray computed tomography (X-ray CT) imaging to observe the amount of resin penetrated into the pores of the aluminum foam. Finally, tensile tests of the obtained samples were conducted to evaluate the bonding strength between aluminum foam and printed resin.

## 2. Materials and Methods

### 2.1. Preparation of Aluminum Foam

The aluminum foam used in this study was fabricated from Al-Si-Cu aluminum alloy ADC12 die-casting sheets by the friction stir welding method [38,39], which is one of the precursor methods. In the precursor method [40,41], solid aluminum mixed with a foaming agent called a precursor is heated to generate pores in the aluminum by the gas generated by the decomposition of the foaming agent, resulting in foaming. In the friction stir welding method, a foaming agent powder is placed between two aluminum sheets and friction stir welding is performed on top of the foaming agent powder to mix the foaming agent powder into the aluminum sheets. The resulting precursor is heated for foaming. The resulting aluminum foam was machined into thin sheets of approximately 5 mm in thickness. Then, the aluminum foam sheet was placed in an electric furnace preheated to 380–400 °C for three hours to fully heat the aluminum foam. In preliminary experiments, when the heating time was longer than 2 h, the entire aluminum foam was sufficiently heated, resulting in a constant temperature of the aluminum foam when it was taken out. In the present study, the holding time was 3 h, taking into account the variability. It took about 1–2 min to take the aluminum foam out of the electric furnace and place it on the 3D printer. In a preliminary experiment, the temperature of the aluminum foam when it was extracted from the electric furnace and placed on the 3D printer was measured using thermocouples. The temperature at the start of printing was 80–200 °C, and the temperature at the end of printing the first layer was about 70–80 °C. Although the temperature at the start of printing varied, the temperature dropped rapidly and the first layer was printed at almost 70–80 °C. Note that the aluminum foam was fabricated by the foaming precursor near the melting point of aluminum. The precursor starts foaming gradually when it is heated above the solidus temperature and foams rapidly above the liquidus temperature [42]. In the case of ADC12, the solidus temperature is 515 °C and the liquidus temperature is 580 °C [43]. Because the heating temperature of the aluminum foam sheet in this study was sufficiently lower than the foaming temperature, no shape changes in aluminum foam, such as pore deformation or re-foaming behavior [44,45], were observed.

### 2.2. The 3D Printing Process

The heated aluminum foam was fixed on the plate of the 3D printer with double-sided tape, and printing was started. Ender3 S1 pro (manufactured by Creality, Shenzhen, China) was used as the 3D printer and Poly-Lactic Acid (PLA) resin was used as a filament. The printed shape was a cylinder with a diameter of 20 mm and a height of 10 mm. This shape was selected to allow the attachment of a round bar jig for tensile testing to evaluate the bonding strength between the aluminum foam and resin after printing. For 3D printing, the thickness of one layer was 0.2 mm, and the printing speed was 60 mm/s. This printing speed was determined by trial and error. If the printing speed was too fast, there were blank areas, and if it was too slow, there were resin puddles; that is, it was not possible to directly print the desired shape accurately either too fast or too slow. The infill density was 20%. However, to allow more resin to penetrate into the pores of the aluminum foam and to firmly attach the tensile test jig, upper and lower layers were printed in a dense layer with an infill density of 100% for a thickness of 0.8 mm (four layers). In order to print with the resin softened as much as possible so that much resin could enter the pores, the printing temperature was set to 230 °C, the highest temperature that could be set by the 3D printer used in this study. To keep the temperature of the aluminum foam at the highest temperature possible during printing, the temperature of the plate was set to 100 °C, the highest temperature that could be set. Furthermore, the 3D printer used in this study could not apply a pushing force during printing. Therefore, the nozzle was placed as close as possible to the aluminum foam so that the resin could easily penetrate into the pores. However, if the nozzle was placed too close to the aluminum foam, the aluminum foam was pulled along by the nozzle during printing and could not be printed on properly, so a gap of about one sheet of paper (0.1 mm thickness) was set. To investigate the effect of preheating of aluminum foam, a sample in which the resin was directly printed on unheated aluminum foam was also fabricated for comparison. In this case, aluminum foam was placed on the 3D printer without heating, and the same printing conditions of the 3D printer as for the heated aluminum foam described above were used. Resin was directly printed on 6 heated aluminum foam samples and 2 unheated aluminum foam samples.

### 2.3. X-ray CT Imaging

The bonded samples were subjected to microfocus X-ray CT imaging (SMX-225CT, Shimadzu Corporation, Kyoto, Japan) to observe the amount of the resin penetrated into the pores of the aluminum foam before the tensile test was conducted. The pixel equivalent length was 50–60 μm, and the slice pitch was the same as the pixel equivalent length.

### 2.4. Tensile Tests

Next, the tensile tests were conducted on the bonded samples to evaluate the bonding strength. A steel round bar with a diameter of 16 mm and a length of 100 mm was attached to the opposite side of the bonding interface using structural adhesive as a tensile jig. Tensile tests were performed with an universal testing machine (Instron 5582, Norwood, MA, USA) at a tensile speed of 1 mm/min. Bonding strength was obtained by dividing the maximum load obtained during the tensile test by the area of the cross-section perpendicular to the tensile direction of the cylindrical resin (the area of a circle 20 mm in diameter).

## 3. Results and Discussion

### 3.1. Obtained Bonded Sample

**Figure 1:**
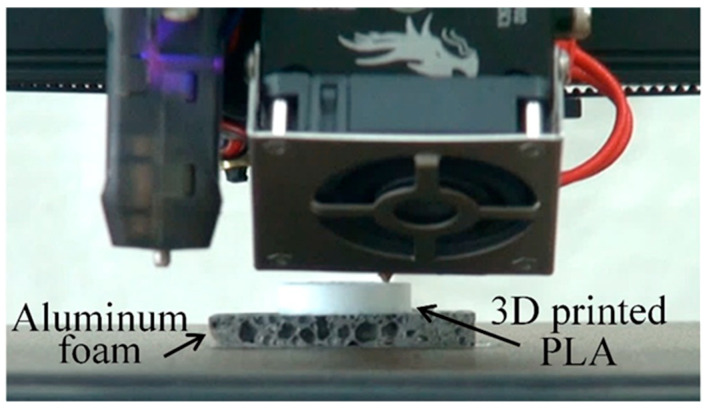
Image of 3D printer printing resin on heated aluminum foam.

Figure 1 shows a 3D printer printing resin on heated aluminum foam. It can be seen that the resin can be directly printed on aluminum foam in the same way as in normal 3D printing. However, if the thickness of the aluminum foam was not uniform and the distance between the nozzle and the aluminum foam surface varied, the aluminum foam would be pulled out at points that were too close and could not be printed on properly. When the thickness of the aluminum foam was uniform, the nozzle position of the 3D printer could be adjusted, even if the thickness varied slightly from sample to sample.

**Figure 2:**
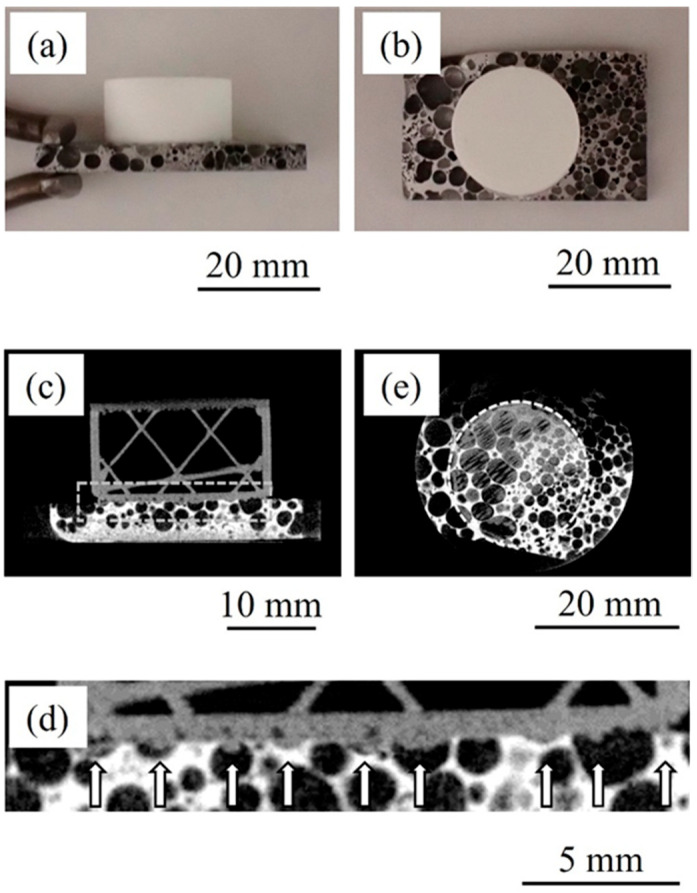
Bonded sample in which resin was directly printed on heated aluminum foam. (**a**) Side view. (**b**) Top view. (**c**) X-ray CT image viewed from side. (**d**) Enlarged image of dotted box in (**c**). White arrows show where the resin penetrated into pores. (**e**) X-ray CT image viewed from above.

Figure 2 shows a bonded sample in which resin was directly printed on heated aluminum foam. Figure 2a,b show a side view and a top view of the sample, respectively. It can be seen that a cylindrical resin can be directly printed on the aluminum foam. The printed samples were bonded and did not separate when removed from the 3D printer. Figure 2c shows a two-dimensional cross-sectional X-ray CT image of the bonded sample viewed from the side. The image was taken at the center of the sample. The lower layer is aluminum foam. The white areas in the aluminum foam are aluminum alloys, and the black areas are pores. The upper layer is printed resin. Figure 2d shows an enlarged view of the area near the bonding interface enclosed by the dotted box in Figure 2c. In the area indicated by the arrow at the bonding interface, resin is observed to have penetrated into the pores. Although there is some variation in the amount of resin that has penetrated, it can be seen that resin can penetrate into almost all the pores. Figure 2e shows a two-dimensional cross-sectional X-ray CT image of the bonded sample viewed from above. The boundary between aluminum foam and resin is observed. The area enclosed by the dotted circle is the printed area. The surrounding pores are observed to be black due to the absence of resin, whereas the gray resin is observed to have penetrated into the pores on the inside, showing that resin can penetrate into almost all the pores. From these results, it is considered that the resin penetrates into the pores and the anchoring effect can be achieved.

**Figure 3:**
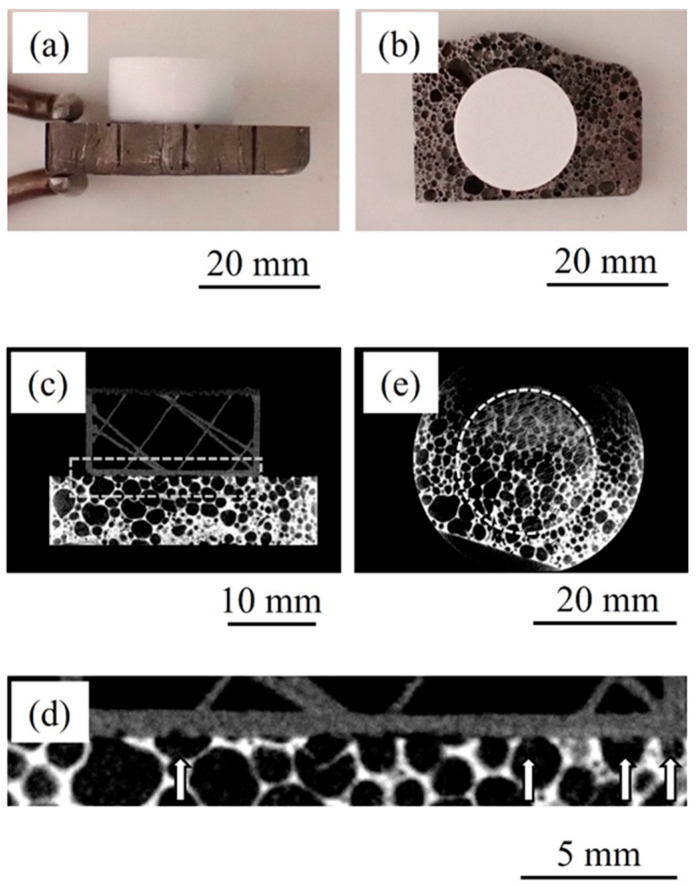
Bonded sample in which resin was directly printed on unheated aluminum foam. (**a**) Side view. (**b**) Top view. (**c**) X-ray CT image viewed from side. (**d**) Enlarged image of dotted box in (**c**). White arrows show where the resin penetrated into pores. (**e**) X-ray CT image viewed from above.

Figure 3 shows a bonded sample in which resin was directly printed on unheated aluminum foam. Figure 3a,b show a side view and a top view of the sample, respectively. It can be seen that a cylindrical resin can be directly printed on the aluminum foam as well as on the heated aluminum foam. The printed samples were bonded and did not separate when removed from the 3D printer. Figure 3c shows a two-dimensional cross-sectional X-ray CT image of the bonded sample viewed from the side. The lower layer shows aluminum foam and the upper layer shows the printed resin. Figure 3d shows an enlarged view of the area near the bonding interface enclosed by the dotted box in Figure 3c. In the area indicated by the arrows at the bonding interface, resin is observed to have penetrated into the pores. However, the amount of penetration is less than in the heated sample, and there are many pores where no resin has penetrated into. Figure 3e shows a two-dimensional cross-sectional X-ray CT image of the bonded sample viewed from above. The boundary between aluminum foam and resin is observed. The area enclosed by the dotted circle is the printed area. This figure also shows that there are only a few pores that have been penetrated by resin and are gray in color. That is, when resin is directly printed on unheated aluminum foam, only a small amount of resin penetrates into the pores, and when the aluminum foam is heated for printing, more resin penetrates into the pores during printing.

### 3.2. Bonding Strength Results

**Figure 4:**
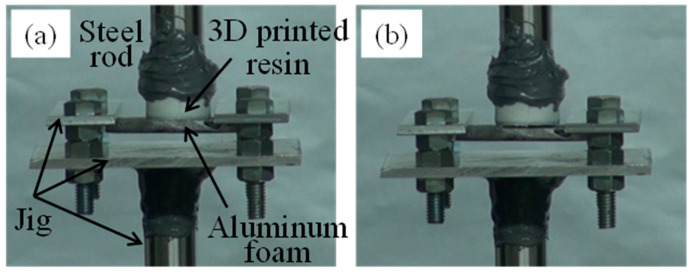
Tensile test of bonded sample in which resin was directly printed on heated aluminum foam. (**a**) Initial state. (**b**) After fracture.

Figure 4 shows a tensile test of the bonded sample in which resin was directly printed on heated aluminum foam. A steel round bar was bonded to the resin on the upper side, and the aluminum foam on the lower side was pulled by a jig made from an aluminum alloy sheet and a round steel bar. Figure 4a shows the initial state before conducting the tensile test and Figure 4b shows the sample after fracture. Fracture occurred at the bonding interface between the resin and aluminum foam. This fracture behavior was similar for both samples directly printed on heated and unheated aluminum foam.

**Figure 5:**
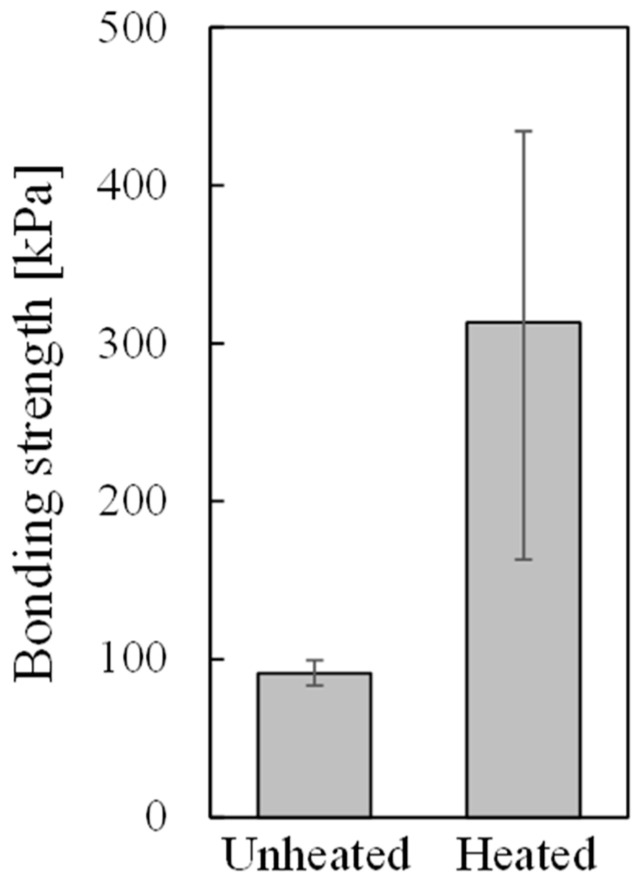
Bonding strengths of samples directly printed on unheated and heated aluminum foam.

Figure 5 shows the average, maximum, and minimum bonding strengths of samples in which resin was directly printed on unheated and heated aluminum foam. Although there is some variation, the sample in which resin was directly printed on the heated aluminum foam shows higher bonding strength.

**Figure 6:**
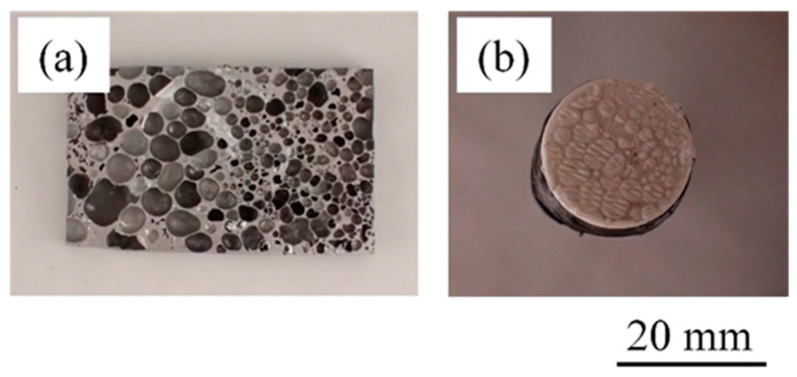
Fracture surfaces after tensile test of bonded sample in which resin was directly printed on heated aluminum foam. (**a**) Aluminum foam side. (**b**) Resin side.

Figure 6 shows the fracture surfaces after the tensile test of the bonded sample in which resin was directly printed on heated aluminum foam. Figure 6a shows the aluminum foam side and Figure 6b shows the resin side. No resin was observed on the aluminum foam side. In addition, the pores did not collapse and maintained their original shape, suggesting that the pores were not damaged during the printing process. Many convexities were observed on the resin side, and the pore shape of the aluminum foam was transferred, suggesting that the resin was pulled out through the pores during the tensile test. This indicates that a large amount of resin penetrated into the pores during printing. These results were similar to the other samples.

**Figure 7:**
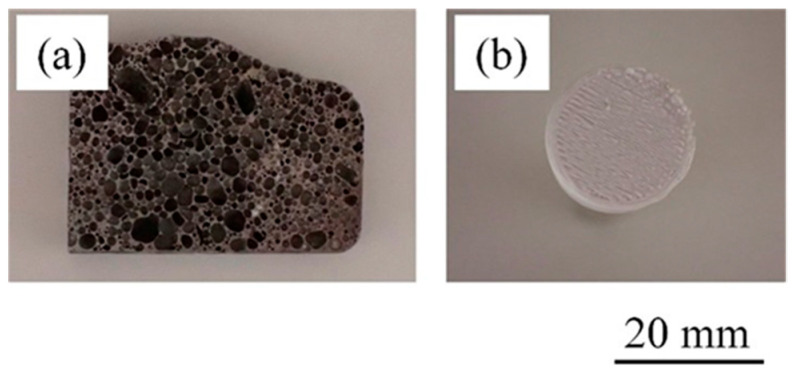
Fracture surfaces after tensile test of bonded sample in which resin was directly printed on unheated aluminum foam. (**a**) Aluminum foam side. (**b**) Resin side.

Figure 7 shows the fracture surfaces after the tensile test of the bonded sample in which resin was directly printed on unheated aluminum foam. Figure 7a shows the aluminum foam side and Figure 7b shows the resin side. No resin was observed on the aluminum foam side, and the pores did not collapse and maintained their original shape, similar to the fractured surfaces shown in Figure 6a. In contrast, although some convexities were observed on the resin side, the heights of the convexities were lower than the sample shown in Figure 6b, indicating that only a small amount of resin penetrated into the pores. Another sample had similar results. These results are consistent with the results of X-ray CT imaging shown in Figure 2 and Figure 3, suggesting that preheating the aluminum foam softens the resin and facilitates its penetration into the pores.

### 3.3. Effect of Amount of Resin Penetration on Bonding Strength

**Figure 8:**
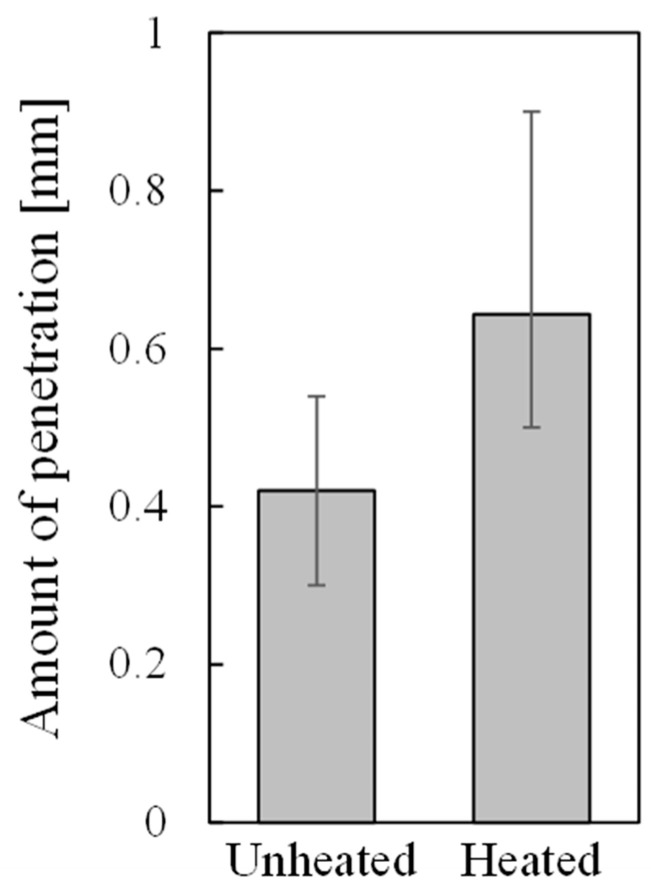
Amount of resin penetration for samples directly printed on unheated and heated aluminum foam.

Figure 8 shows the average of the amount of penetration for all samples, and the maximum and minimum amount of resin penetration for those samples that showed the maximum and minimum amount of resin directly printed on unheated and heated aluminum foam. The amount of resin penetration into the pores was obtained by using X-ray CT images parallel to the bonding interface, such as Figure 2e and Figure 3e. The amount of resin penetration was defined as the number of X-ray CT images in which resin was visible multiplied by the slice pitch of X-ray CT images. Although the amount of resin penetration varied from place to place even in a single specimen, it was obtained based on the number of X-ray CT images in which resin was visible even slightly. It can be seen that more resin penetrates into the samples in which resin was directly printed on heated aluminum foam than the samples in which resin was directly printed on unheated aluminum foam. This suggests that heating aluminum foam for printing softens the resin and allows more resin to penetrate into the pores during printing. This large amount of penetration is considered to cause a high anchor effect; therefore, higher bonding strength was obtained for the sample in which resin was directly printed on heated aluminum foam than for the sample in which resin was directly printed on unheated aluminum foam as shown in Figure 5.

**Figure 9:**
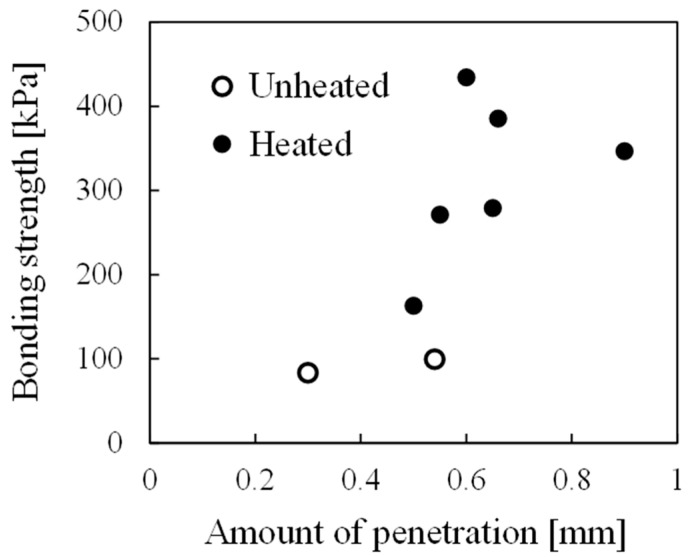
Relationship between bonding strength and amount of resin penetration into pores.

Figure 9 shows the relationship between the bonding strength and the amount of resin penetration into the pores. Although there was some variation, the bonding strength tended to be higher for samples with a larger amount of resin penetration. One possible reason for this variation is the variety in the pore structures of aluminum foam. The amount of resin penetration is small in pores that were originally small. It is also assumed that some pores are easily penetrated by resin, while others are difficult to penetrate, depending on the pore size. It is well known that variations in pore size can occur even within a single aluminum foam sample when it is fabricated by the foaming process, and these variations in pore structures are considered to have influenced the variations in bonding strength. In addition, variations in pore size result in some pores being easy to penetrate and others being difficult to penetrate, even within a single sample. The bonding strength is considered to be higher when a large amount of resin penetrates into a large number of pores, and this is also considered to be one of the causes of the variation.

Note that when printing on a flat aluminum plate without holes, the printing itself was completed, but it was not bonded and peeled off immediately. To increase the amount of resin penetration into pores, it is necessary to keep the temperature of the aluminum foam a little higher during printing. It is assumed that the softening of the resin would continue for a longer period of time and more resin would penetrate into the pores at a higher temperature of the aluminum foam. This problem can be solved by introducing a heater on the 3D printer that can reach higher temperatures, which will be further investigated in the future.

**Figure 10:**
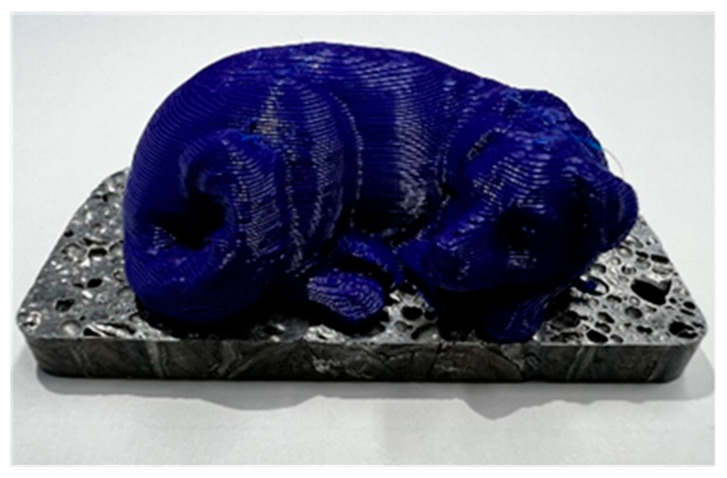
Sleeping dog directly printed on aluminum foam.

In this study, it was found that the 3D printer can directly print resin on aluminum foam with a certain degree of high bonding strength. By using a 3D printer, it is expected that a resin with a complex shape such as the sleeping dog shown in Figure 10 can be combined with aluminum foam, allowing the desired product shape to be realized freely.

This direct printing method is expected to be able to 3D print with other resins as well. Although optimization of the preheating temperature is necessary for each resin used, it is assumed that bonding can be achieved in the same way if the resin is allowed to enter the pores.

## 4. Conclusions

In this study, the fabrication of composite materials by directly printing resin on an aluminum foam surface using a 3D printer was attempted. The resin was directly printed on both heated and unheated aluminum foam. The results obtained are shown below:

(1) It was shown that composite materials consisting of aluminum foam and resin can be fabricated by directly printing resin with a 3D printer on both heated and unheated aluminum foam.
(2) It was shown that the resin was softened during the printing process in the case of directly printed resin on heated aluminum foam, allowing more resin to penetrate into the pores than in the case of directly printed resin on unheated aluminum foam.
(3) It was shown that resin can be directly printed on the aluminum foam with a high bonding strength, as a large amount of resin penetrates into the pores, resulting in an anchor effect. That is, composite materials consisting of aluminum foam and arbitrary-shaped resin with relatively high bonding strength can be fabricated when a large amount of resin is allowed to penetrate into the pore.

## Data Availability

Data are contained within the article.
